# Mathematical dynamic integration model based on game theory and optimization theory

**DOI:** 10.1371/journal.pone.0361429

**Published:** 2026-10-29

**Authors:** Jing Wang, Xuanwei Huang, Shuai Li

**Affiliations:** 1 School of Information Engineering, Jiangxi Polytechnic University, Jiujiang, China; 2 Supervision and Inspection Office of Discipline Inspection Commission, Jiangxi Polytechnic University, Jiujiang, China; 3 School of Mathematics and Information Science, Nanchang Hangkong University, Nanchang, China; Beijing Institute of Technology, CHINA

## Abstract

As technology advances, the research on mathematical dynamic integration models has yielded a potent instrument for the analysis and optimization of complex systems, thereby fostering progress across multiple domains. To solve the issues of traditional dynamic integration models, such as the lack of solid mathematical theoretical support, imbalanced weight allocation among multiple participants, and insufficient dynamic adaptability, this study integrates the Shapley value method from cooperative game theory with Monte Carlo sampling techniques to construct an information evaluation model. The calculation of the marginal contributions of participants in different alliances enables the quantification of the value of multi-source information, thus resolving the issue of imbalanced weight allocation. Furthermore, a dynamic optimization model is built by integrating Nash equilibrium and Markov decision processes, utilizing state transition probabilities to capture temporal changes in strategies and achieve stable strategy optimization. Finally, a mathematical model that includes integration is designed. Experiments that compare the two models show that the average time it takes to make a decision goes up by only 0.2 seconds when the number of samples is between 25 and 150, and the amount of memory used goes up by only 1.2GB. In the InfiMM-WebMath-40B dataset, the model’s standard deviation increases from 0.020 in 25 samples to 0.030 in 150 samples, an increase of only 0.010. In the MV-MATH dataset, the standard deviation increases by only 0.011. Furthermore, the proposed model outperforms the comparative models in all metrics, reducing convergence time by 14.7%, training time by 15.3%, energy consumption by 16.3%, and iteration count by 21.5%, while maintaining a low central processing unit utilization rate. Therefore, the proposed mathematical dynamic integration model can more accurately handle uncertain information in complex systems, optimize system decisions, reveal the inherent laws governing dynamic changes in systems, and provide stronger theoretical support and methodological guidance for solving practical problems.

## 1 Introduction

Bioinformatics, epidemiology, social sciences and other sreas need to process data from different sources and at different time scales. Mathematical models can integrate this information, provide a unified perspective, and thus understand complex systems and provide decision support [1,2]. To effectively manage complex systems, it is necessary to establish accurate and practical mathematical models. However, existing models often suffer from problems such as information asymmetry, multi-objective conflict and computational complexity. They lack effective tools to quantify the contributions and synergistic effects of participants, leading to weight imbalances—such as over-reliance on highly reliable participants and neglecting potentially high-value information [3]. Furthermore, a large amount of redundancy is generated in information transmission, which not only increases computational costs but also interferes with decision-making, such as redundant resource allocation calculations caused by repeated demand statistics [4].

Game theory is a theoretical framework for studying how participants maximize their individual interests through strategy selection under given rules [5,6]. Y. Yan’s team proposed a path planning model based on game theory to solve potential safety risks in intelligent transportation. This model combines non-cooperative game theory with local cooperative game theory to predict vehicle driving behavior. Experimental findings denote that the model has good applicability in various lane-changing scenarios [7]. In the area of communication, to improve communication quality, S. Fu et al. integrated game theory with deep learning to construct a communication resource allocation model based on non-cooperative game theory. This model utilizes deep neural networks to dynamically predict vehicle mobility and channel state, thereby achieving efficient and low-latency data transmission. Simulation experiments denote = that this model can significantly improve network throughput and communication reliability in high-density urban traffic environments, while effectively reducing system energy consumption [8]. To solve the dilemma faced by tactical communication environments, X. Lin et al. proposed an interaction framework between servers and edge nodes based on game theory. Simulation results show that the proposed framework has significant performance [9].

Optimization theory can provide a mathematical framework for information filtering and reasoning path optimization. For example, information bottleneck theory can compress redundant information, and Markov Decision Process (MDP) optimization can improve planning efficiency [10]. For example, V. Goyal et al. proposed to use a probability transition factor model to optimize MDP to improve the robustness of MDP. Experiments denoted that the proposed method reduces the uncertainty of MDP parameters and has strong effectiveness [11]. A. Heidari et al. proposed to use MDP for optimization in response to the problem of insufficient computing power in Internet of Things, and then constructed a task offloading scheme. Experimental denote that the proposed scheme can effectively reduce latency, reduce energy consumption and task failure rate [12]. S. S. Shinde et al. proposed an MDP-based optimization scheme in response to the limited resources of vehicle edge computing servers. Simulation results show that the scheme can reduce energy consumption and thus effectively improve the performance of vehicle networks [13].

Traditional dynamic ensemble methods rely heavily on empirical rules and lack a solid mathematical theoretical foundation. A single model often only reflects local characteristics of a system, failing to fully grasp its complexity [14,15]. Therefore, this study proposes a mathematical dynamic ensemble model that integrates a Shapley-value-based information evaluation model with an MDP-based Nash Equilibrium (NE) optimization model, aiming to provide a unified mathematical framework for dynamic ensembles. The innovation of this method lies in dynamically combining game theory and optimization theory to achieve synergy between equilibrium and optimization objectives, thereby overcoming the problem of static models’ inability to adapt to environmental changes and providing more practical theoretical and algorithmic support for decision-making in complex systems.

## 2 Methods and materials

### 2.1 Information evaluation model based on Shapley value method

Ordinary models have deficiencies in dealing with issues such as information asymmetry and interference. Game theory offers a powerful analytical framework for strategic interactions in competitive and cooperative environments, and can handle cooperative and competitive relationships among multiple agents. It is an ideal tool for evaluating the value of participants [16]. The Kernel method aims to maximize the league’s minimum residual and is unique; however, since it involves solving a linear programming problem, it has high computational complexity, and the computational overhead increases significantly when there are a large number of participants. The weighted Banzhaf value calculates the key voting contributions of each entity; while it is faster than the Kernel method, it does not satisfy the validity axioms of the Shapley value, resulting in an allocation where the total contributions do not match the system’s total payoffs. The Shapley value method in cooperative games constructs a global explanatory framework. By considering the contribution of each feature in different modes to allocate the model output, it helps to understand the model behavior more clearly, thereby supporting more informed decision-making [17,18]. Therefore, this study introduces the Shapley value method to fairly allocate the total payoff generated by the alliance composed of all participants. The process of the Shapley value method is shown in Fig 1.

**Fig 1:**
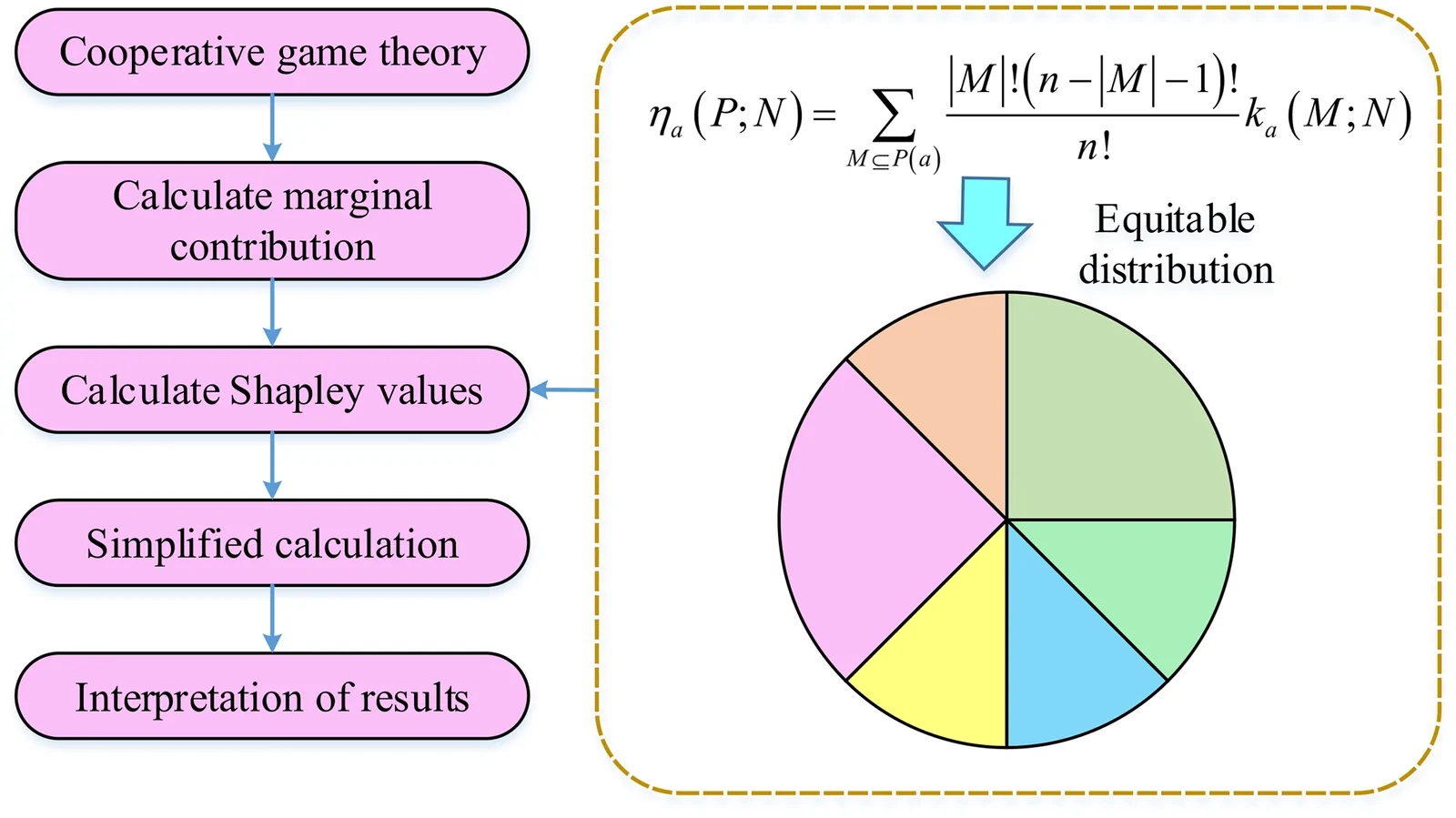
Shapley value method flowchart.

As shown in Fig 1, the Shapley value method first defines the game problem, clarifies the set of participants, and sets a characteristic function to describe the total payoff of different combinations of participants. Then, the method evaluates the marginal payoff brought by each participant when joining all possible combinations of participants (i.e., alliances), combines the corresponding weight coefficients with the fairness principle of participant ranking, and performs a weighted sum of all marginal contributions of each participant to determine their contribution [19]. The calculation of the Shapley value considers all possible orders of participants joining the alliance, ensuring the fairness of the calculation results. Finally, a value reflecting the average marginal contribution is calculated for each participant, resulting in an allocation scheme, thereby fairly allocating information value to each participant [20]. The formula for calculating the marginal contribution is shown in Equation (1).


$$\begin{array}{c} k_{a}\left(M;N\right)=N\left(M\cup \left\{a\right\}\right)-N\left(M\right) \end{array}$$ (1)

In Equation (1), $k_{a}\left(M;N\right)$ represents the marginal contribution of participant a between information sets M and N, i.e., the contribution of participant a to the increase in total information content after joining information set M; N(M∪{a}) represents the information content of information set M after adding participant a; N(M) represents the information content of information set M itself, i.e., the total information content without participant a. Then, the model calculates the Shapley value of each participant to determine which participants or participants contribute the most to the information value, as shown in Equation (2).


$$\begin{array}{c} \eta_{a}\left(P;N\right)=\sum_{M\subseteq P\left(a\right)}\frac{\left|M\right|!\left(n-\left|M\right|-1\right)!}{n!}k_{a}\left(M;N\right) \end{array}$$ (2)

Equation (2), $\eta_{a}\left(P;N\right)$ is the Shapley value of the participant a in the information evaluation model; n! is the number of permutations of all participants; |M| is the number of participants in the subset M. Then, the expected contribution of the participant w to the accuracy of the system decision in all possible cases is calculated, as shown in Equation (3).


$$\begin{array}{c} N_{w}\left(Q\right)=\sum_{a\in \left[L\right]}Pr\left(g^{\prime }=a,g=a\right) \end{array}$$ (3)

In Equation (3), $N_{w}\left(Q\right)$ is the Shapley value of participant w under model Q, i.e., the contribution of participant w to the whole system; $g^{\prime }$ and g are the optimal decision and the actual decision, respectively; L is the total number of participants. Then, the decision accuracy is improved by participant w, as shown in Equation (4).


$$\begin{array}{c} M_{Q_{a};w;B}=\eta_{Q_{a}}\left(Q;N_{w}\right) \end{array}$$ (4)

In Equation (4), $M_{Q_{a};w;B}$ represents the Shapley value for a specific model $Q_{a}$, participant w, and dataset B, i.e., the contribution of participant w to model $Q_{a}$ on dataset B. The contribution of each participant to the entire model is then evaluated, as shown in Equation (5).


$$\eta_{Q_{a}}\left(Q;N_{w}\right)=\sum_{M\subseteq P\left(a\right)}\frac{\left|M\right|!\left(n-\left|M\right|-1\right)!}{n!}\left(N_{w}\left(M\cup \left\{a\right\}\right)-N_{w}\left(M\right)\right)$$ (5)

Equation (5) is obtained by considering all possible combinations of participants, calculating the marginal contribution of each participant w in each combination, and then taking a weighted average of these marginal contributions. The formula for calculating the marginal contribution is shown in Equation (6).


$$\begin{array}{c} F_{A;w;B}=\eta_{A}\left(Q;N_{w}\right)-\sum_{Q_{a}\in A}\eta_{Q_{a}}\left(Q/A\cup Q_{a};N_{w}\right) \end{array}$$ (6)

In Equation (6), $F_{A;w;B}$ represents the marginal contribution of participant w to the model set A and the dataset B. Finally, the Shapley value is standardized to measure the average marginal contribution of participants to the model performance and is normalized for comparison, as shown in Equation (7).


$$\begin{array}{c} {M^{\prime }}_{Q_{a};w;B}=\frac{\text{1}}{\lambda_{w}}\cdot \eta_{Q_{a}}\left(Q;N_{w}\right) \end{array}$$ (7)

In equation (7), $M_{Q_{a};w;B}$ is the standardized Shapley value under model $Q_{a}$, participant w and dataset B; $\lambda_{w}$ is the normalization factor used to standardize the Shapley value. In the evaluation of multi-subject, high-dimensional information, the Shapley value method has the problem that the amount of computation increases exponentially with the number of subjects. Monte Carlo sampling can reduce redundant computation and improve efficiency by random sampling [21]. Therefore, this study uses Monte Carlo sampling to quantify the impact of uncertainties such as subject weight ambiguity and information value fluctuation on the Shapley value results, so as to make the evaluation more comprehensive. The operation of Monte Carlo sampling is shown in Fig 2.

**Fig 2:**
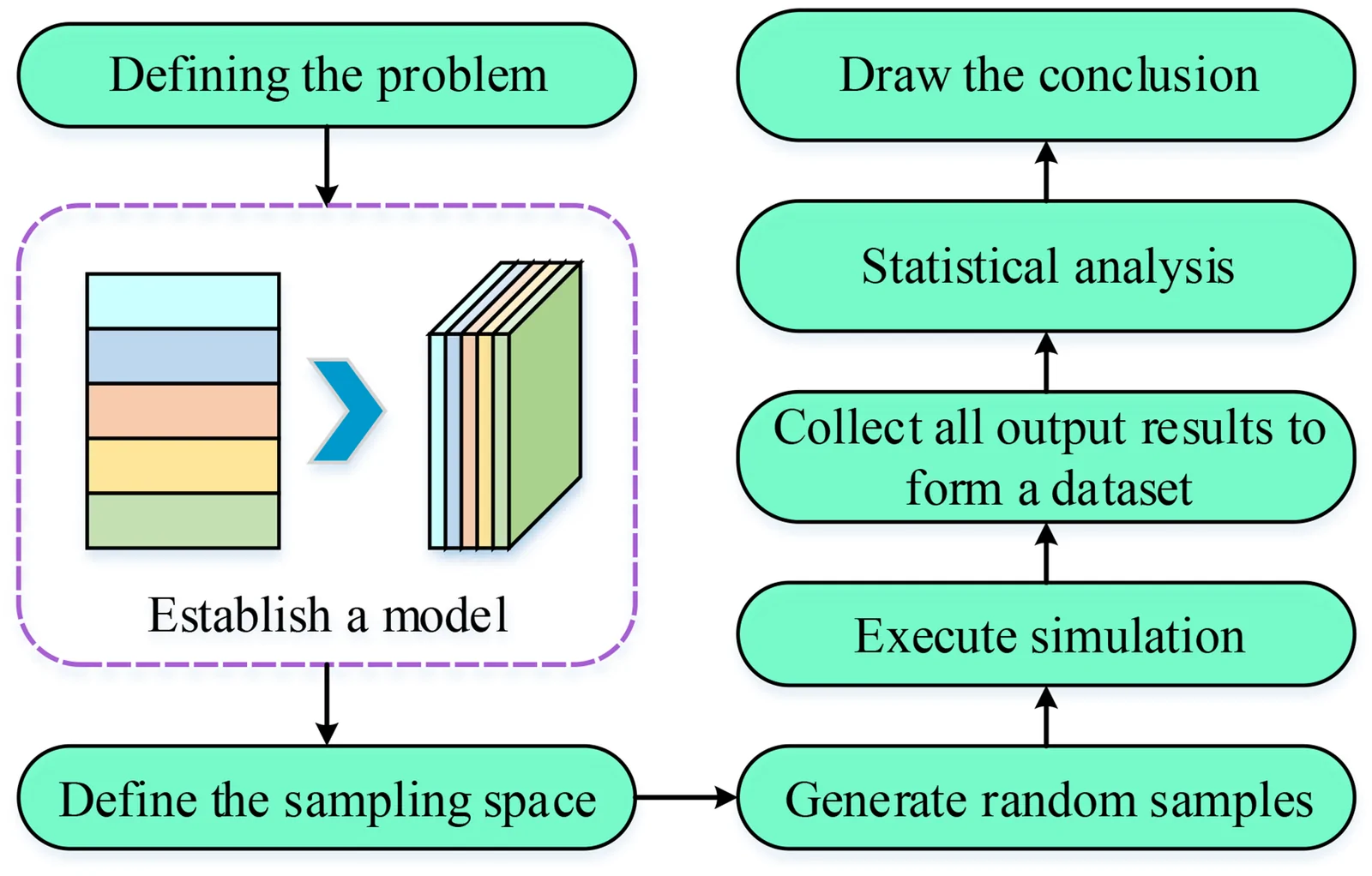
Flowchart of Monte Carlo sampling.

As shown in Fig 2, Monte Carlo sampling generates a large number of random samples within a certain range and uses the statistical properties of these samples to estimate the target value. In practical problems, due to the real-time nature and complexity of the problem, it is difficult to obtain an exact solution directly through analytical methods. Monte Carlo sampling provides an effective approximate solution approach [22]. Monte Carlo sampling first performs real-time estimation of noise intensity for input information from multiple sources, calculating the residual variance and confidence interval width for each information source as metrics for quantifying noise. Lower and upper bounds are set for the sample size to prevent it from shrinking indefinitely or expanding without limit. When the input noise intensity is detected to be low and the confidence interval for the Shapley value estimate has converged within the preset error threshold, the Monte Carlo sample size is reduced, the number of coalition combination samples is decreased, computational overhead is minimized, and inference speed is ensured; When noise intensity increases and the confidence interval for the estimate exceeds the permissible error threshold, the sample size is automatically increased, the number of random coalition samples is raised, the variance of the sampling estimates is suppressed, and the accuracy of the Shapley value weight assessment is ensured. At the same time, an iteration termination criterion is set: if the relative change in the Shapley value after two consecutive sampling rounds is less than a given threshold, sampling is terminated early to further conserve computational resources. Although Monte Carlo sampling alleviates the exponential computational burden associated with Shapley values, the overall computational cost still rises significantly as the number of agents grows, making it difficult to scale to complex systems involving a vast number of participants. Consequently, this research introduces a sparse sampling mechanism to optimize the sampling space. Based on a pre-screening process using normalized Shapley values, low-value agents with contributions below a preset threshold are excluded, and marginal contribution calculations are performed only for the subset of high-contribution agents. For the remaining low-contribution agents, a grouped aggregation sampling approach is employed, merging multiple low-weight agents into virtual coalition units to reduce the number of coalition combinations requiring evaluation. This strategy sparsifies the participant set during the sampling phase, thereby reducing the size of the sampling space while ensuring that key informational contributions are preserved. Therefore, the operation of the information evaluation model is shown in Fig 3.

**Fig 3:**
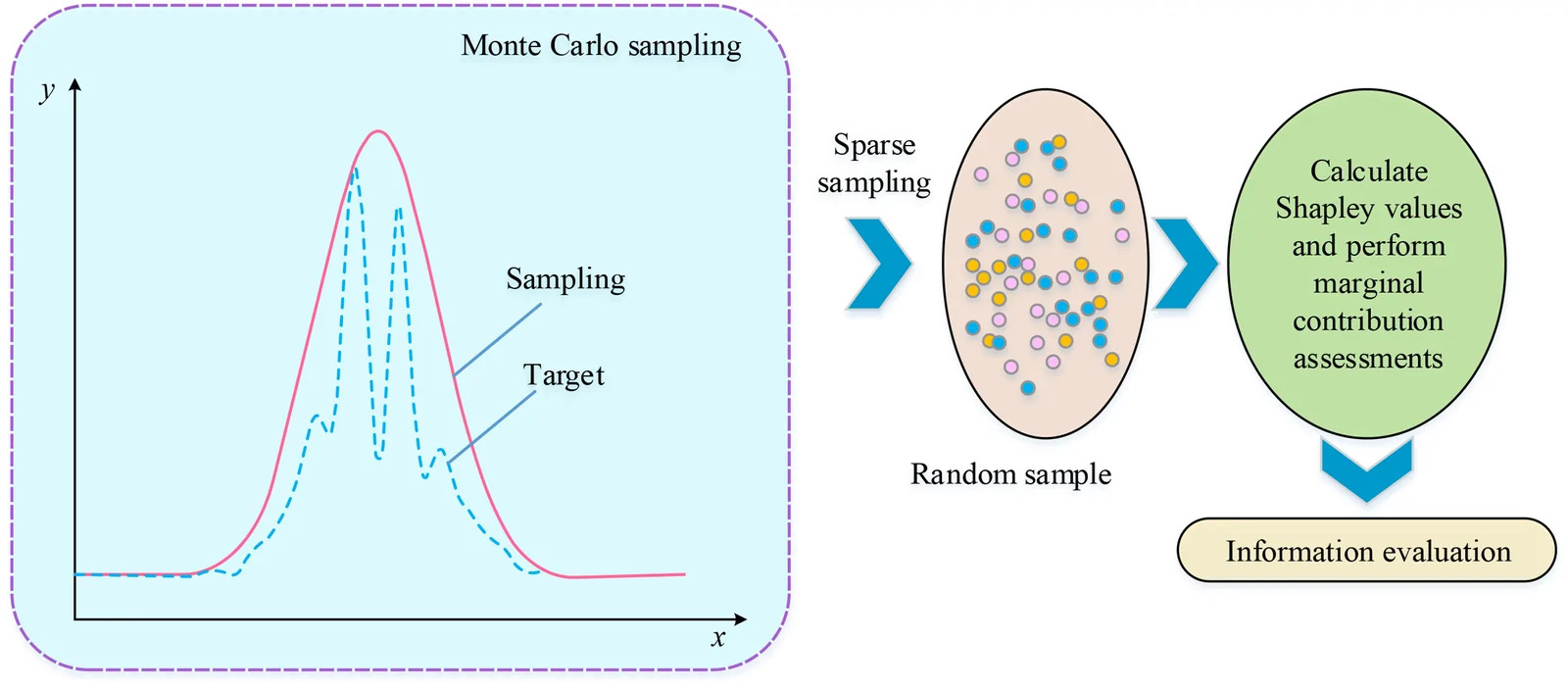
Information e valuation model operational diagram.

As shown in Fig 3, the model first clarifies the core objectives and key influencing indicators of information assessment. It then simulates the dynamic probability distribution and uncertainty scenarios of information elements and related variables through Monte Carlo sampling to generate a sufficient sample dataset that conforms to reality. Based on the fair allocation characteristics of the Shapley value method in game theory, the model combines the sample data to quantify the marginal contribution value and global importance weight of each information element to the assessment objective, thereby achieving a reasonable decomposition of information value. Finally, it integrates the weight results and sampling scenario analysis to output the comprehensive assessment results and uncertainty boundaries of the information, thus realizing information assessment.

### 2.2 Mathematical dynamic integration model based on game theory and optimization theory

The information evaluation model based on the Shapley value method achieves fair and accurate value allocation by quantifying the marginal contribution of each information source, providing a core basis for the screening and priority ranking of multi-source information. However, this method has inherent limitations in dynamic scenarios. It relies on the computational logic of a fixed alliance structure, which is difficult to cope with the system state iteration and decision target adjustment requirements brought about by environmental changes, and lacks the ability to dynamically adapt to the strategy evolution process. The mathematical modeling of complex systems often needs to realize the closed loop of “evaluation-decision-optimization” in a dynamic environment. Simple information evaluation cannot support the real-time adjustment of strategies and the continuous adaptation of targets. Therefore, the study introduces a dynamic optimization framework and determines a set of strategy combinations through NE, so that any party can not improve its own income by changing its strategy alone, thus avoiding the continuous oscillation of the model in the iteration [23]. The principle of NE is shown in Fig 4.

**Fig 4:**
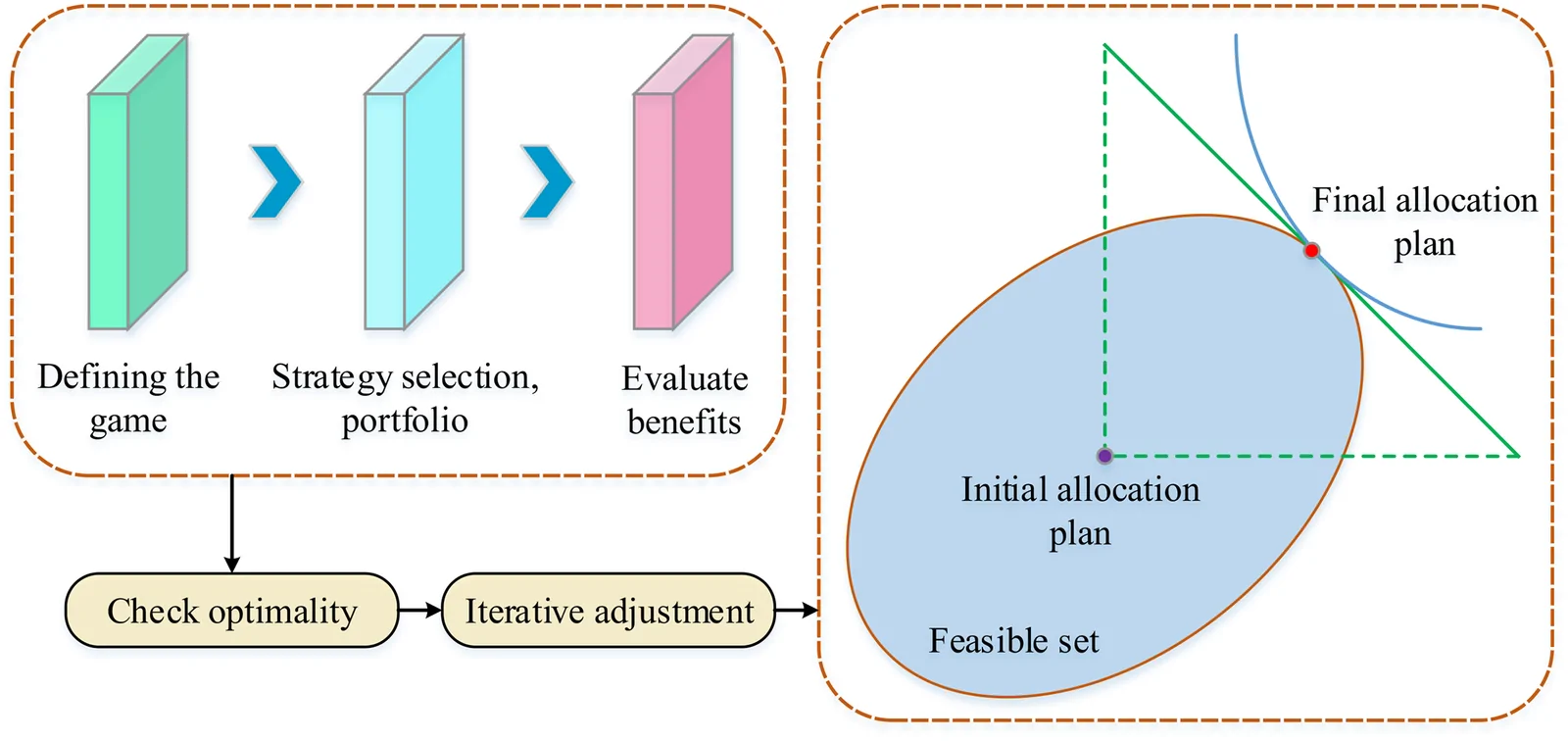
NE s chemical diagram.

As shown in Fig 4, firstly, the participants in the game, the strategies that each participant can choose, and the corresponding payoffs are clearly defined; then, it is assumed that all participants are rational individuals who will choose strategies based on the principle of maximizing their own payoffs; then, it is determined whether there exists a strategy combination in which any single participant cannot increase their payoffs by changing their own strategy when the strategies of other participants are fixed. If such a strategy combination exists, then the strategy combination achieves NE [24]. NE finds stable strategies through inequalities, as shown in Equation (8).


$$z_{c}\left(e_{c}^{*},E_{-c}^{*}\right)\geq z_{c}\left(e_{c},E_{-c}^{*}\right)$$ (8)

In Equation (8), $z_{c}$ is the value function associated with participant c; $e_{c}^{*}$ and $e_{c}$ are the optimal and arbitrary policies of participant c, respectively; $E_{-c}^{*}$ is the optimal policy of all participants except participant c. In the NE state, when participant c adopts his optimal policy $e_{c}^{*}$, he will at least not gain less utility compared to all other participants adopting their optimal policies $E_{-c}^{*}$. The calculation formula for the optimal policy is shown in Equation (9).


$$\begin{array}{c} e_{c}^{*}\in arg\underset{e_{c}\in E_{c}}{max}z_{c}\left(e_{c},E_{-c}^{*}\right),c=1,2,\cdots ,m \end{array}$$ (9)

In Equation (9), argmax is the parameter that makes the function reach its maximum value; m is the number of participants. Through equation (9), each participant finds its corresponding optimal strategy, and thus reaches a stable state, namely NE. In practical applications, NE is found or approximated through symmetry, thereby reducing the amount of computation, as shown in Equation (10).


$$\begin{array}{c} z_{c}\left(e_{c},e_{d},E_{-c-d}\right)=z_{d}\left(e_{d},e_{c},E_{-c-d}\right) \end{array}$$ (10)

In Equation (10), $E_{-c-d}$ is the set of strategies of the participants other than c and d. Assuming that the participants’ utility functions are continuous and the MDP state space is finite, according to the NE existence theorem, this game has at least one mixed-strategy Nash equilibrium. Applying the MDP value iteration operator T to the value function v, the operator satisfies the contraction mapping condition, as shown in Equation (11).


$$\begin{array}{c} ∥Tv_{\text{1}}-Tv_{\text{2}}|{|}_{\infty }\leq \gamma ||v_{\text{1}}-v_{\text{2}}|{|}_{\infty } \end{array}$$ (11)

In Equation (11), $v_{\text{1}}$ and $v_{\text{2}}$ are the joint value functions of two arbitrary participants, representing the state values corresponding to different strategy combinations in the game; γ is the discount factor, and γ∈(0,1) is used to apply a decay weight to future game payoffs, ensuring that long-term returns are bounded. NE mainly focuses on static equilibrium. In a dynamic environment, the policies of the participants may change over time. NE cannot capture such dynamic changes, while MDP describes the changes in the system state through state transition probabilities and can handle policy selection and optimization in dynamic environments [25]. Therefore, this study uses MDP to optimize NE. The operation of MDP is shown in Fig 5.

**Fig 5:**
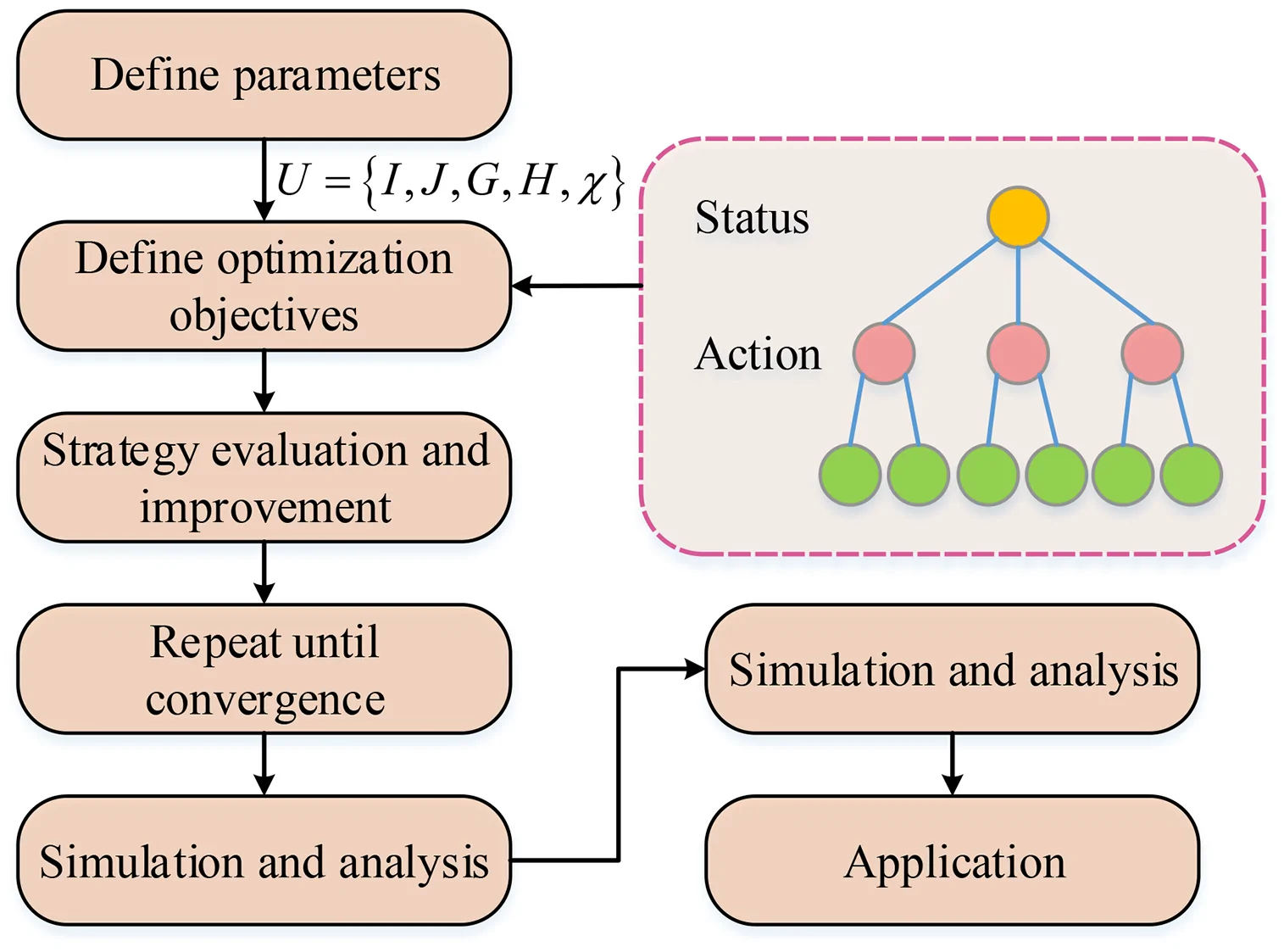
MDP operational c hart.

As shown in Fig 5, in the initial state, the agent selects an action based on the current state and the preset strategy. This action will cause the system to move to the next state and obtain the corresponding immediate benefit. After entering the new state, the agent ignores the historical state information and repeats the cycle of “selecting an action based on the current state→moving to the next state and obtaining the benefit”. The whole process continues until the termination state is reached. Finally, by optimizing the strategy, the agent maximizes the cumulative benefit obtained in the long-term cycle [26,27]. The model predicts the probability of the next state based on the current state and the action taken, as shown in Equation (12).


$$\begin{array}{c} G_{ii^{\prime }}^{j}=G\left(I_{t+1}=i^{\prime }|I_{t}=i,J_{t}=j\right) \end{array}$$ (12)

In Equation (12), $G_{ii^{\prime }}^{j}$ is the probability of transitioning from state i to state $i^{\prime }$ when taking action j; $I_{t+1}$ is the system state at time t+1; and $J_{t}$ is the action taken at time t. State transition probabilities are estimated and derived primarily through two methods: First, historical statistical estimation, which relies on the system’s existing operational history samples to calculate the frequency with which the system transitions to the next state after executing a corresponding action in the current state, using this frequency as the empirical transition probability. This method is suitable for scenarios with sufficient historical observation data; Second, a driven derivation based on standardized Shapley information weights is employed. The contribution weights of each information source are treated as state features and fed into the model. By leveraging the tendency of high-contribution information sources to influence state evolution, the original empirical transition counts are weighted and corrected, thereby yielding updated transition probabilities. Furthermore, to address situations where insufficient samples result in zero observations for certain state-action combinations, a Dirichlet prior is introduced to perform Bayesian smoothing. This avoids the issue of transition probabilities being set to zero and ensures that the overall transition matrix satisfies the normalization constraints of non-negative probabilities and a row sum of 1. Then, the probability of transitioning from state i to state $i^{\prime }$ under a given policy y is calculated, as shown in Equation (13).


$$\begin{array}{c} G_{ii^{\prime }}^{y}=\sum_{j\in J}y\left(j|i\right)G_{ii^{\prime }}^{j} \end{array}$$ (13)

In Equation (13), y(j|i) is the probability of taking action j in state i. Given policy y, the expected value i of the future reward expected to be obtained by taking action j in state i is calculated as shown in Equation (14).


$$\begin{array}{c} H_{i}^{j}=V\left[H_{t+1}|I_{t}=i,J_{t}=j\right] \end{array}$$ (14)

In Equation (14), V is the expected value operator, used to calculate the expected return under given conditions. Then, the long-term average rate of return $\gamma_{y}\left(x\right)$ starting from the initial state x under the given strategy y is calculated, and the performance of different strategies in the long run is evaluated, as shown in Equation (15).


$$\begin{array}{c} \gamma_{y}\left(x\right)=\underset{n\to \infty }{lim}\frac{\text{1}}{n}V\left[\sum_{y=0}^{n}H\left(X_{y},j_{y}\right)|X_{\text{0}}=x\right] \end{array}$$ (15)

In Equation (15), $\frac{\text{1}}{n}$ represents the average of the total reward over each time step; $X_{\text{0}}$ represents the initial state. In summary, the calculation formula for the optimal strategy is shown in Equation (16).


$$\begin{array}{c} \gamma_{y^{\prime }}\left(x\right)=\underset{y}{max}\gamma_{y}\left(x\right) \end{array}$$ (16)

In Equation (16), $\gamma_{y^{\prime }}\left(x\right)$ represents the maximum long-term average return that can be obtained by adopting the optimal strategy under the initial state x. Therefore, the study shows that introducing MDP can better handle problems such as dynamic environment, incomplete information and computational complexity, thereby constructing a more effective optimization model. The process of the NE-MDP optimization model is shown in Fig 6.

**Fig 6:**
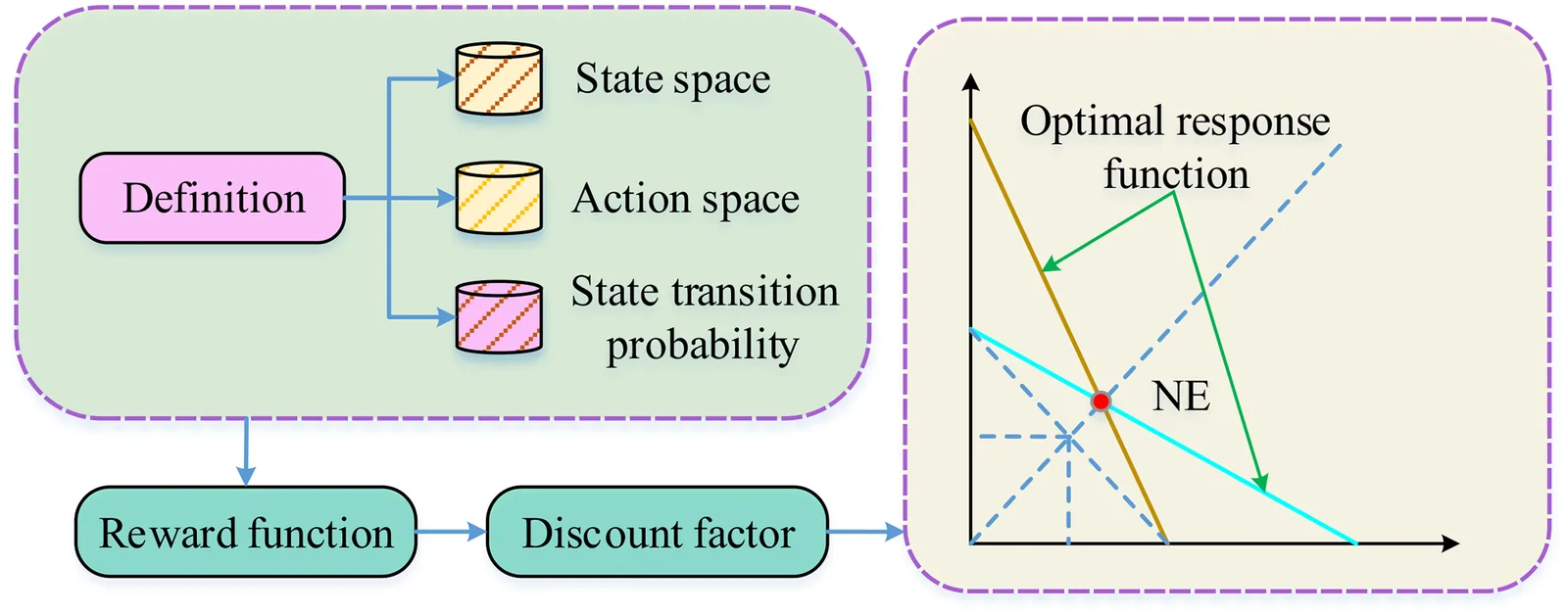
NE – MDP optimization model flowchart.

As shown in Fig 6, the model first determines the state space, action space, and state transition probabilities for each participant. Then, it calculates the expected utility by analyzing the optimal strategy for each participant under different states. Next, dynamic programming is used for strategy iteration, which evaluates and updates the strategy to find the strategy that maximizes the long-term average return. Complex system decision-making (such as bioinformatics data analysis, epidemiological resource allocation, and multi-agent collaboration in social sciences) is a core component of efficient operation in various fields. The accurate quantification of information value and dynamic optimization of strategies have significant theoretical and practical value for improving the scientific nature of decision-making, reducing system operating costs, and coping with environmental uncertainties. In complex system decision-making, multi-source information often exhibits characteristics such as uneven value distribution, strong dynamism, and high redundancy, and is influenced by multiple factors including participant strategy games and time-varying environmental states. Traditional dynamic integration methods, lacking a solid mathematical theoretical foundation, struggle to balance the fairness of information evaluation with the stability of strategy optimization, thus limiting the efficiency and robustness of decision-making. Mathematical dynamic integral models have not only promoted the theoretical development of mathematics and related disciplines but also provided effective solutions to complex practical problems. Therefore, this study integrates game theory and optimization theory to overcome the limitations of traditional single theories. The mathematical dynamic integration model based on Shapley-NE-MDP is denoted in Fig 7.

**Fig 7:**
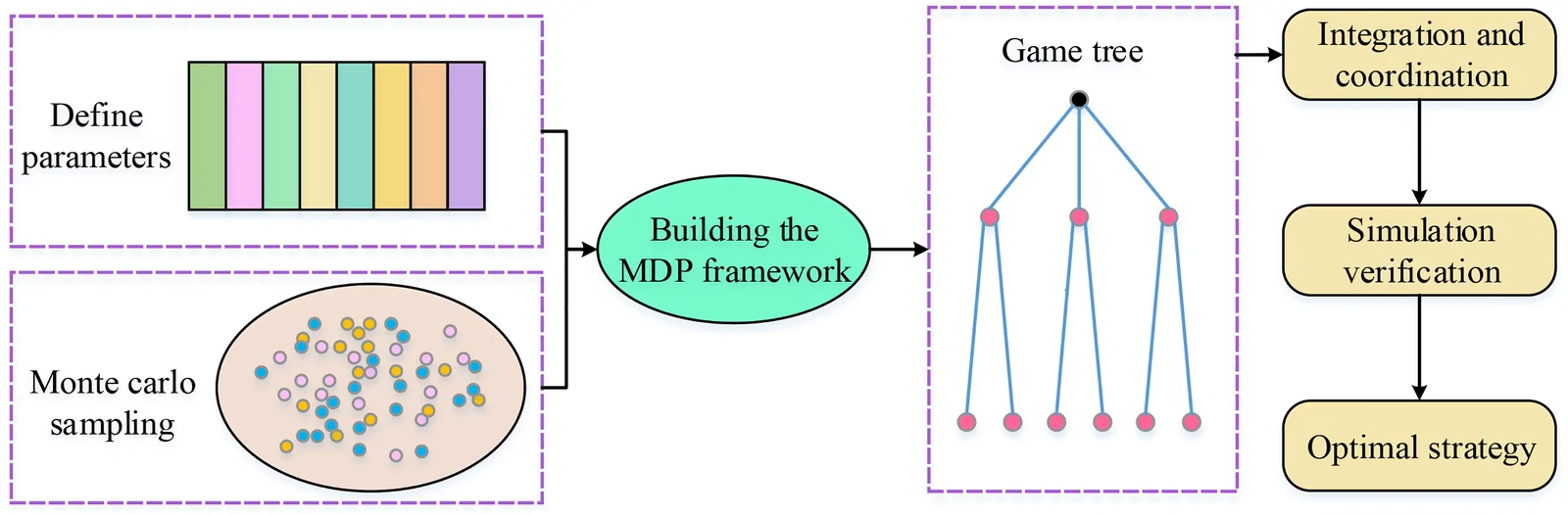
Mathematical dynamic integration model operation diagram.

In Fig 7, the Shapley value is first utilized to assess each participant’s contribution to the total utility of the system, guaranteeing that each participant receives a fair reward. Then, based on the predicted state transition probabilities and their own utility function, each participant selects the optimal policy to maximize their expected utility through the MDP framework. In this process, participants consider the policies that other agents may adopt, and adapt to the dynamic environment through iteration and learning, ultimately reaching a NE state, thereby achieving optimal integration and coordinated operation of the entire system.

## 3 Results

### 3.1 Performance analysis

To assess the effectvieness of the Shapley-NE-MDP model, the InfiMM-WebMath-40B dataset (a multimodal mathematical reasoning dataset that gathers a large amount of mathematical and scientific knowledge, including text, formulas, symbols, and images) and the CORD-19 dataset were employed in the experiment. Eighty sets of samples were randomly selected, and Shapley-NE, NE-MDP, Stackelberg-MDP, and Q-Learning models were selected for comparative experiments. The accuracy, Mean Absolute Percentage Error (MAPE), Root Mean Squared Error (RMSE), F1 score, and recall of the models were recorded, as denoted in Table 1.

**Table 1:** Performance comparison of models.

Models	InfiMM-WebMath-40B					CORD-19				
	Accuracy	MAPE	RMSE	F1	Recall	Accuracy	MAPE	RMSE	F1	Recall
Shapley-NE-MDP	92.3%	3.8%	2.1	0.91	90.5%	87.5%	5.6%	3.0	0.85	84.9%
Shapley-NE	78.6%	8.5%	5.7	0.76	75.2%	71.9%	11.9%	7.9	0.68	67.6%
NE-MDP	85.1%	5.2%	3.6	0.83	82.7%	78.4%	7.7%	5.3	0.75	74.3%
Stackelberg-MDP	81.4%	6.7%	4.3	0.79	79.1%	74.6%	9.1%	6.0	0.71	71.0%
Q-Learning	76.2%	9.3%	6.2	0.73	72.8%	69.3%	12.4%	8.1	0.65	64.8%

As shown in Table 1, on the InfiMM-WebMath-40B dataset, the Shapley-NE-MDP model achieved an accuracy of 92.3%, significantly outperforming other models. This is followed by NE-MDP (85.1%) and Stackelberg-MDP (81.4%), while Shapley-NE (78.6%) and Q-Learning (76.2%) had relatively lower accuracy. Shapley-NE-MDP demonstrated the most optimal MAPE at a mere 3.8%, signifying its capacity for superior error control. Conversely, Q-Learning exhibited the highest MAPE of 9.3%, indicating a comparatively substantial error margin. The Shapley-NE-MDP model demonstrated the lowest RMSE of 2.1, indicating its superior performance in this regard. The recall rate of 90.5% for this model was the highest of all models examined. In addition, the Shapley-NE-MDP algorithm demonstrated a superior F1 score of 0.91, further underscoring its efficacy. At the same time, the Shapley-NE-MDP model exhibited the smallest performance drop, with its accuracy decreasing from 92.3% on the InfiMM-WebMath-40B dataset to 87.5% on the CORD-19 dataset, demonstrating the stability of cross-domain transfer. The data comparison demonstrates that the Shapley-NE-MDP model performs best in all five metrics: accuracy, MAPE, RMSE, F1 score, and recall, indicating the most significant performance.

To further validate the stability of the Shapley-NE-MDP model, the experiment used Shapley-NE-MDP, Shapley-NE, NE-MDP, Stackelberg-MDP, and Q-Learning models. 140 sets of samples were randomly selected from the InfiMM-WebMath-40B dataset and the MV-MATH dataset, respectively, to test the average number of iterations of the model, as shown in Fig 8.

**Fig 8:**
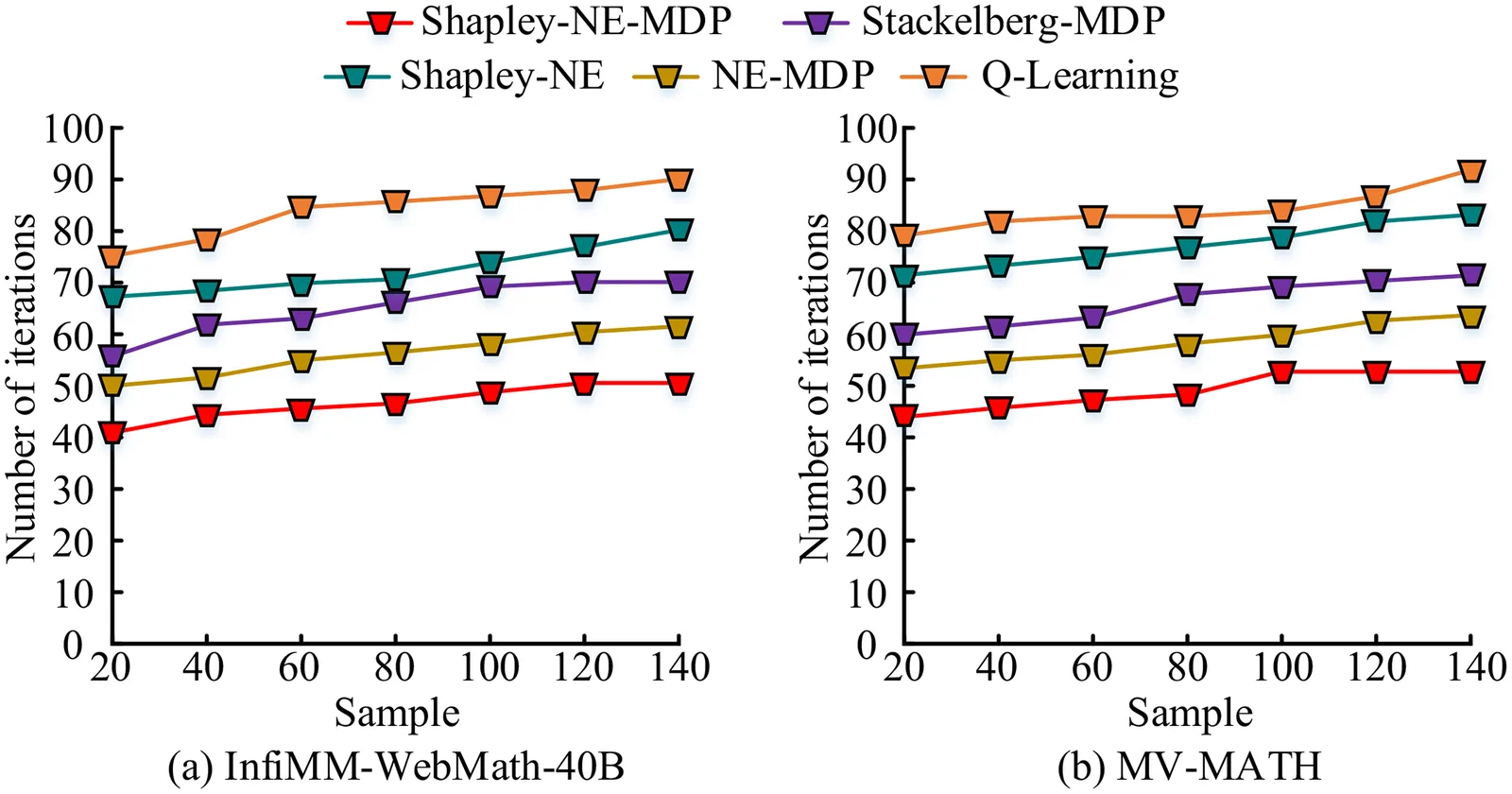
Changes in the number of iterations for the model.

In Fig 8(a), on the InfiMM-WebMath-40B dataset, the number of iterations of the Shapley-NE-MDP model slowly increased from 41 to 50, with a small increase in the curve, making it the model with the lowest and most stable number of iterations. The number of iterations of Q-Learning started from 75 and continued to increase to 90, with a clear upward trend in the curve, and the number of iterations remained at the highest level. The number of iterations of the Shapley-NE model increased from 67 to 80, with a gentle upward trend in the curve. The number of iterations of the NE-MDP model gradually increased from 50 to 61, with a steady upward trend in the curve. The number of iterations of the Stackelberg-MDP model increased by 14. In Fig 8(b), the number of iterations for Shapley-NE-MDP increased from 44 to a stable 53, similar to its performance on the InfiMM-WebMath-40B dataset. The number of iterations for the Q-learning model steadily rose from 79 to over 90, maintaining its position as the model with the highest number of iterations and demonstrating a clear upward trend. Meanwhile, Shapley-NE increased from around 71–83 iterations, displaying a gentle upward curve. NE-MDP increased from 54 to around 63 iterations, showing a stable upward trend. Meanwhile, the Stackelberg-MDP model fluctuated between 60 and 72 iterations, exhibiting a trend similar to that observed in the InfiMM-WebMath-40B dataset. Experiments demonstrate that the Shapley-NE-MDP model exhibits stronger adaptability and greater stability across different data scales.

To test the applicability of the Shapley-NE-MDP model, experiments were performed using Shapley-NE-MDP, Shapley-NE, NE-MDP, Stackelberg-MDP, and Q-Learning models. 150 sets of samples were randomly selected from the InfiMM-WebMath-40B dataset to test and record the average inference time (s) and memory usage (GB) of the models, as denoted in Fig 9.

**Fig 9:**
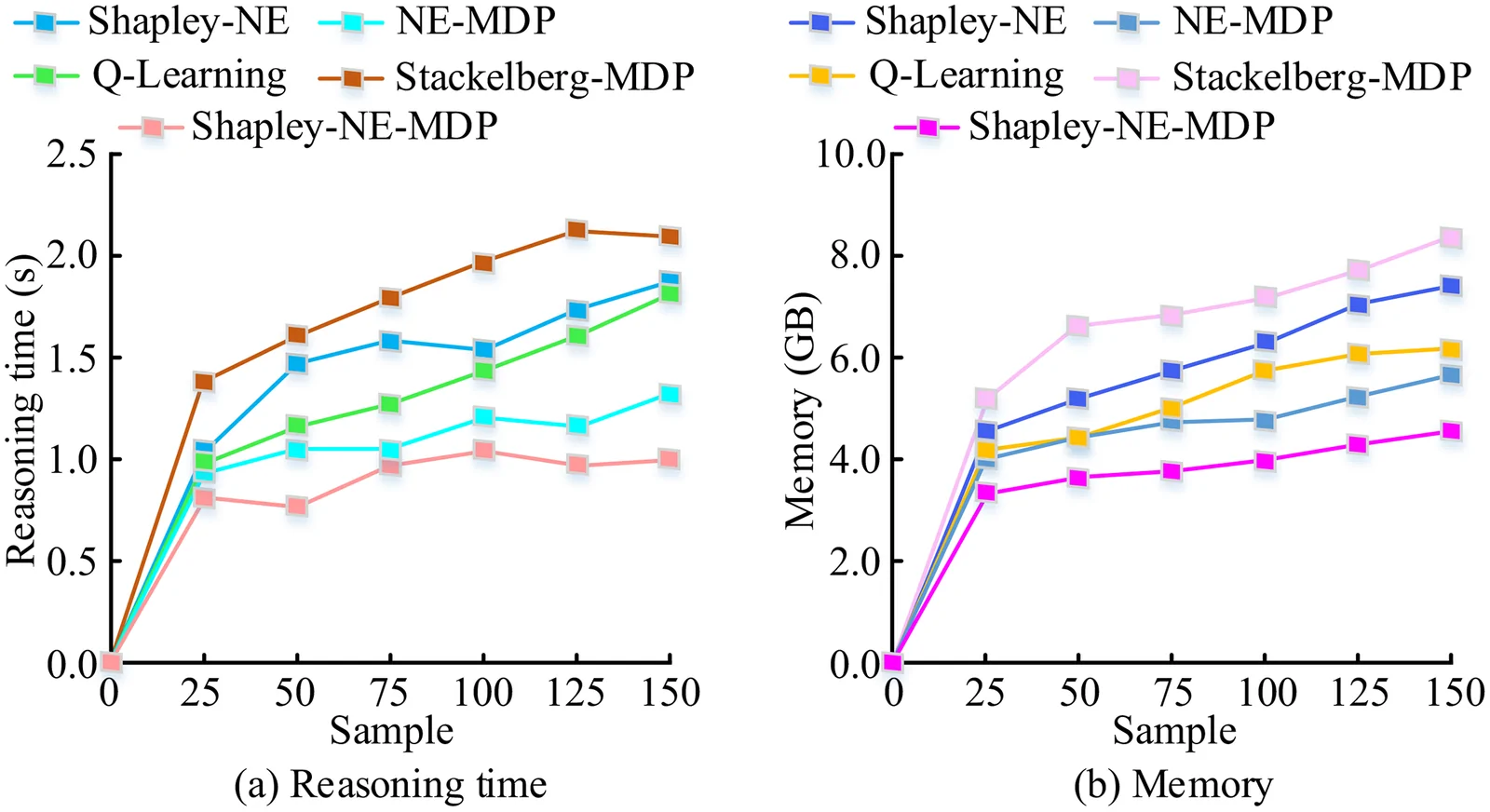
Changes in model inference time and usage memory.

In Fig 9(a), the inference time of the Shapley-NE-MDP model was 0.8s with 25 samples, and then stabilized at 1.0s, making it the shortest and most stable model, demonstrating high inference efficiency. The Stackelberg-MDP model’s inference time continued to increase to 2.1s, with the curve showing a clear upward trend, and the inference time remained at the highest level. The Shapley-NE model slowly increased to 1.9s, with the curve showing an upward trend. The Q-Learning model increased to about 1.8s, with the curve rising steadily. The NE-MDP model remained relatively stable when it increased to 1.0s, and then rose slightly to about 1.3s, with the curve being relatively flat. In Fig 9(b), the memory usage of Shapley-NE-MDP was approximately 3.4 GB with 25 samples, then steadily increased to around 4.6 GB, showing the lowest memory usage and the most gradual increase among all models, demonstrating good memory efficiency. Stackelberg-MDP ‘s memory usage continued to increase to 8.4 GB, with a clear upward trend, consistently remaining at the highest level. Shapley-NE ‘s memory usage slowly increased to 7.4 GB, with a gently rising curve. Q-Learning gradually increased to 6.2 GB, with a stable upward curve. NE-MDP slowly increased to around 5.7 GB, with a relatively flat curve. The experimental results indicate that the Shapley-NE-MDP model exhibits the best efficiency stability and resource adaptability when the sample size is expanded, and has stronger generalization ability.

To further verify the generalization ability of the Shapley-NE-MDP model, Shapley-NE, NE-MDP, Stackelberg-MDP, and Q-Learning were selected as comparison models. 150 sets of samples were randomly selected from the InfiMM-WebMath-40B dataset and the MV-MATH dataset for repeated experiments, and the standard deviation of the models was calculated, as shown in Fig 10.

**Fig 10:**
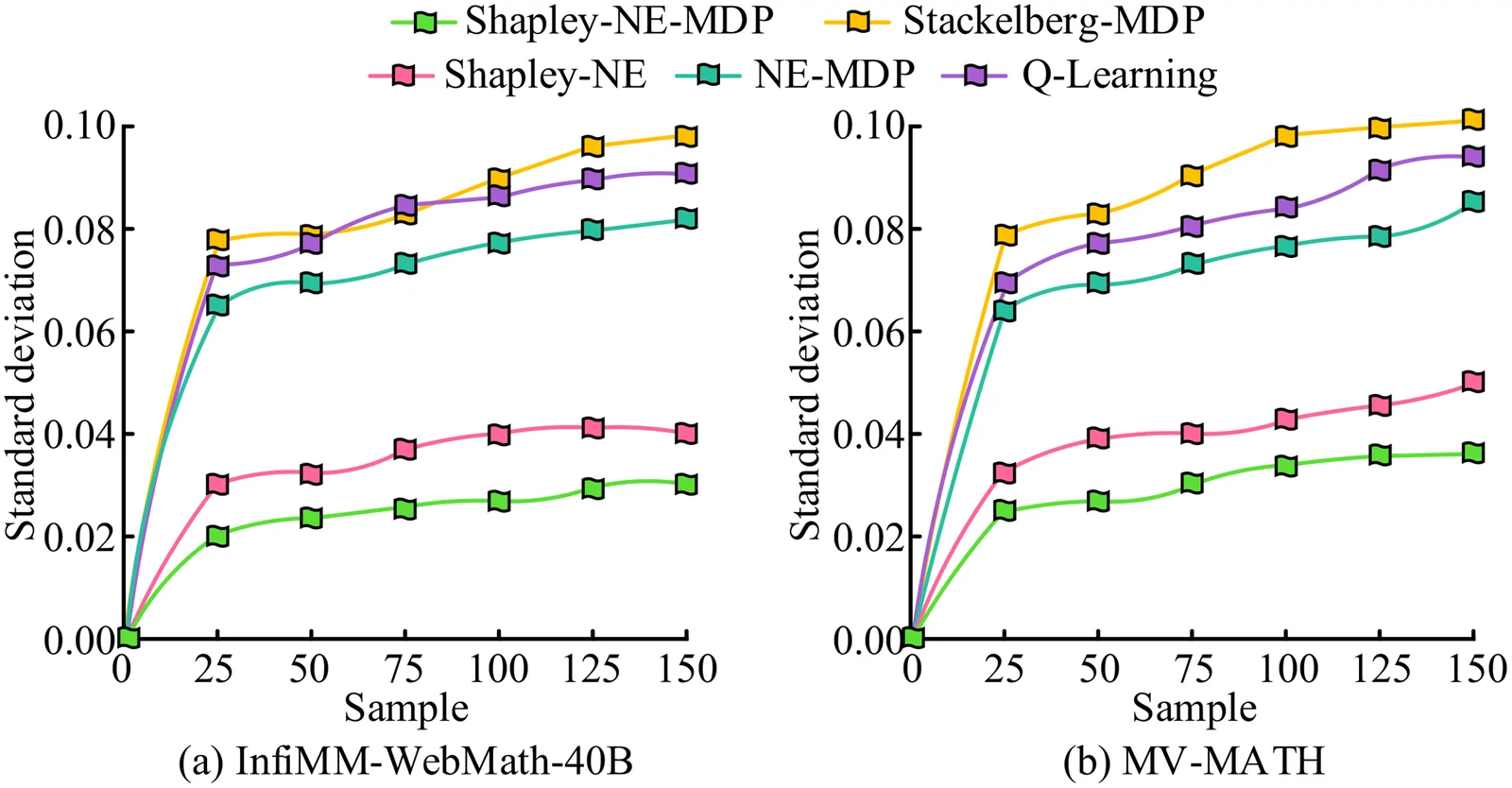
Variation in the standard deviation of the model.

In Fig 10(a), the standard deviation of the Shapley-NE-MDP model was 0.020 with 25 samples, and then increased slowly to 0.030 with 150 samples, which is the smallest and the most gradual increase among all models. The standard deviation of the Stackelberg-MDP model was 0.078 with 25 samples, and continued to rise, reaching close to 0.100 with 150 samples, remaining at the highest level and exhibiting the worst stability. The Q-Learning model gradually increased to 0.091, with a gentle upward curve. The NE-MDP model increased steadily to 0.082. The Shapley-NE model increased slowly to 0.040, showing relative stability. In Fig 10(b), the standard deviation of Shapley-NE-MDP was approximately 0.025 with 25 samples, then steadily increased to approximately 0.036 with 150 samples. Stackelberg-MDP continued to climb to 0.100, remaining the model with the highest standard deviation, indicating insufficient stability. Q-Learning gradually increased from 0.070 with 25 samples to 0.094 with 150 samples, showing a gentle upward curve. NE-MDP steadily increased to 0.085. Shapley-NE slowly increased to 0.050, showing relative stability. Experiments show that the Shapley-NE-MDP model has the smallest standard deviation on both datasets, and its curve shows the gentlest growth, indicating that the Shapley-NE-MDP model has the best output stability and the strongest generalization ability across data scales and scenarios.

### 3.2 Application analysis

To verify the effectiveness of the Shapley-NE-MDP-based mathematical dynamic ensemble model in practical applications, comparative experiments were conducted using Shapley-NE, NE-MDP, Stackelberg-MDP, Q-Learning, and Shapley-NE-MDP-based mathematical dynamic ensemble models. The convergence time (s), training time (s), energy consumption (Wh), CPU utilization, and number of iterations of each model were tested under 110 sets of data, as shown in Table 2.

**Table 2:** Analysis of the model’s application effectiveness.

Models	Convergence time	Training time	Energy	CPU usage	Number of iterations
Shapley-NE-MDP	87.3	156.8	12.8	78.5	426
Shapley-NE	124.6	213.5	18.6	82.3	678
NE-MDP	102.4	185.2	15.3	80.1	543
Stackelberg-MDP	141.7	246.9	21.4	85.7	752
Q-Learning	136.2	238.7	20.1	84.2	715

As shown in Table 2, the Shapley-NE-MDP model had the fastest convergence time of 87.3s, followed by NE-MDP (102.4s), Shapley-NE (124.6s), and Q-Learning (136.2s). Stackelberg-MDP had the longest convergence time (141.7s); Shapley-NE-MDP had a training time of 156.8s, exceeding the training efficiency of NE-MDP (185.2s), Shapley-NE (213.5s), and Q-Learning (238.7s), while Stackelberg-MDP had the longest training time at 246.9s; Shapley-NE-MDP had the best energy efficiency with a power consumption of only 12.8Wh; Shapley-NE-MDP had the lowest CPU utilization at 78.5%, accounting for only 98.0%, 95.4%, and 93.2% of NE-MDP (80.1%), Shapley-NE (82.3%), and Q-Learning (84.2%), respectively, while Stackelberg-MDP had the highest CPU utilization at 85.7%. At the same time, Shapley-NE-MDP had the highest iteration efficiency with 426 iterations. Experimental results show that the Shapley-NE-MDP model has higher efficiency, lower resource consumption, and stronger performance in practical applications, and is more suitable for deployment in scenarios that are sensitive to time, energy consumption, and hardware resources.

To verify the robustness of the Shapley-NE-MDP-based mathematical dynamic integration model, a comparative experiment was conducted by adding Gaussian noise of different intensities (low, medium, and high). The accuracy reduction of the model in 280 samples was calculated, as shown in Fig 11.

**Fig 11:**
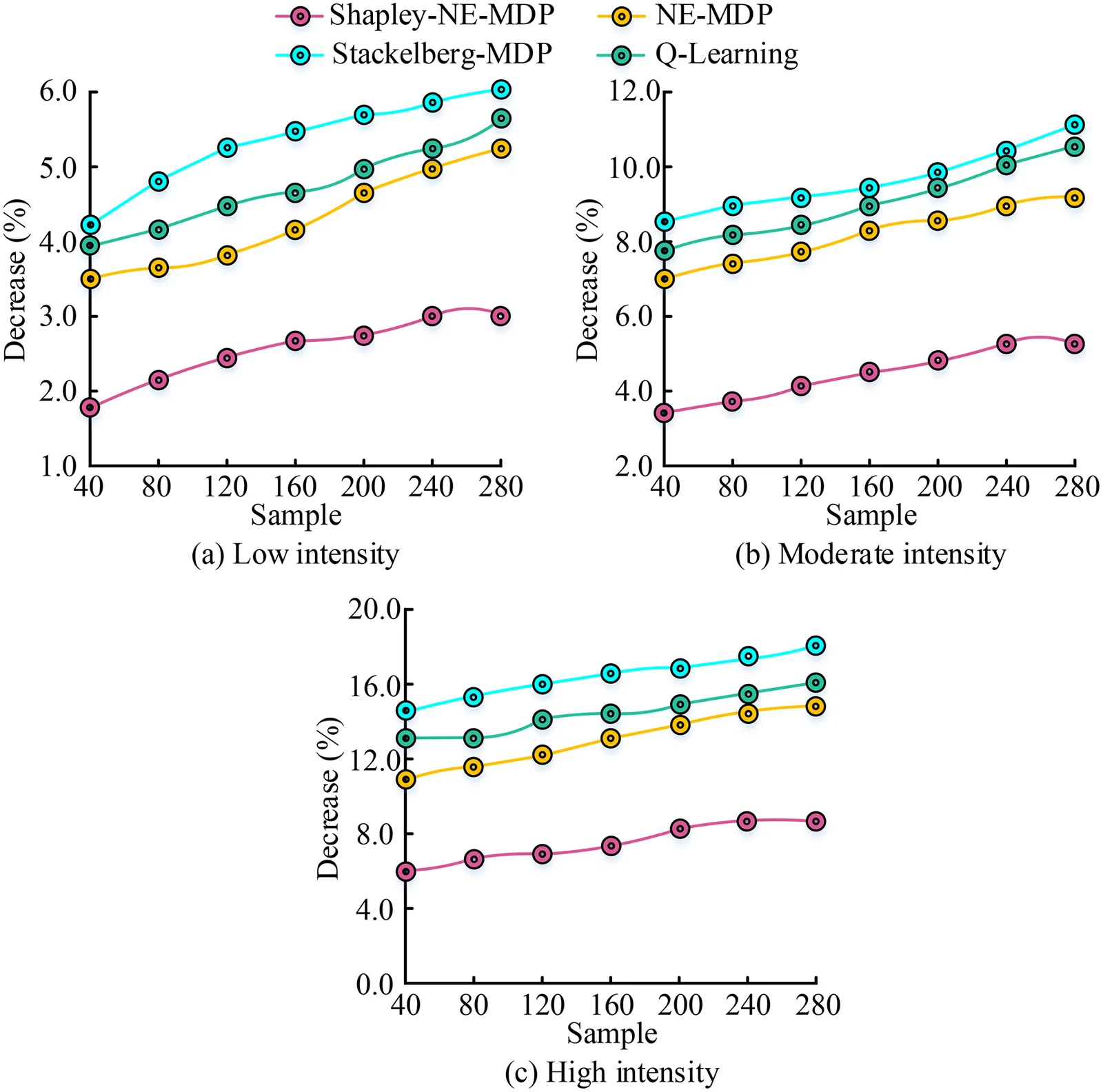
Comparison of accuracy decline in models under different noise levels.

In Fig 11(a), the accuracy of the Shapley-NE-MDP model in low-intensity Gaussian noise environment increased from 1.8% to 3.0%, an increase of 1.2%, which is the smallest increase among the models; NE-MDP increased from 3.5% to 5.2%, with a steady upward curve; Q-Learning increased from 4.0% to 5.6%, an increase of 1.6%, showing a clear upward trend; Stackelberg-MDP increased from 4.2% to 6.0%, an increase of 2.8%, with the largest decrease. In Fig 11(b), Shapley-NE-MDP increased from 3.4% to 5.2%, a growth of 1.8%, still the model with the smallest growth rate, demonstrating significant robustness; NE-MDP increased from 7.0% to 9.2%, a growth of 2.2%, with a steady upward curve; Q-Learning increased from 7.8% to 10.6%, a growth of approximately 2.8%, showing a clear upward trend; Stackelberg-MDP increased from 8.6% to 11.2%, a growth of 2.6%. In Fig 11(c), the accuracy decrease of the Shapley-NE-MDP model slowly increased from 6.0% to approximately 8.8%, maintaining the smallest increase even under high-intensity noise. The NE-MDP model increased from 10.8% to 14.8%, a 4.0% increase, with a smooth upward curve. Q-Learning increased from 13.2% to 16.0%, a 2.8% increase, showing a clear upward trend. The Stackelberg-MDP model increased from 14.8% to 18.0%, a 3.2% increase. The experimental results show that the Shapley-NE-MDP model exhibits the smallest increase in accuracy decrease under low, medium, and high-intensity Gaussian noise, indicating its strong robustness.

To verify the applicability of the mathematical dynamic integration model based on Shapley-NE-MDP, the models were grouped according to the difficulty of the mathematical problems (calculus/differential equations). Basic arithmetic was used as the reference benchmark. The sensitivity of the mathematical dynamic integration models based on NE-MDP, Stackelberg-MDP, Q-Learning, and Shapley-NE-MDP was calculated on 140 sets of sample data ((accuracy of low difficulty – accuracy of high difficulty)/ accuracy of low difficulty × 100%), as shown in Fig 12.

**Fig 12:**
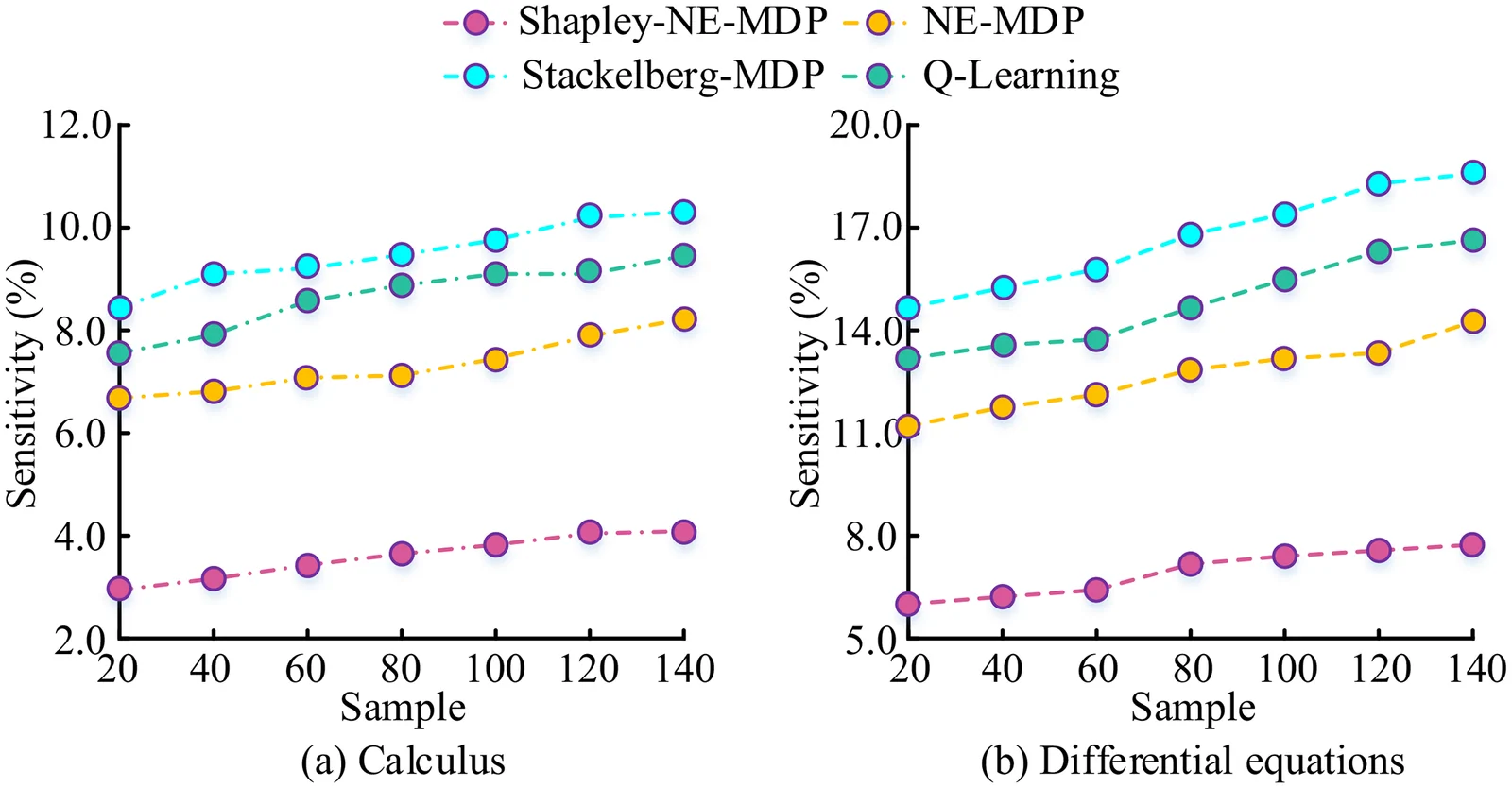
Sensitivity analysis of the models.

In Fig 12(a), as the sample size increased from 20 to 140, the sensitivity of all models showed an upward trend. Q-Learning increased from 7.6% to 9.4%, an increase of 1.8%; NE-MDP increased from 6.6% to 8.2%; the Stackelberg-MDP model had a sensitivity of 8.4% with a sample size of 20, which increased to 10.3% with a sample size of 140, an increase of 1.9%; the Shapley-NE-MDP model’s sensitivity slowly increased from 3.0% to about 4.0%, which was 5.4% lower than Q-Learning, 4.2% lower than NE-MDP, and 6.3% lower than Shapley-NE-MDP. In Fig 12(b), the Stackelberg-MDP sensitivity was approximately 14.6% with a sample size of 20 and approximately 18.5% with a sample size of 140; Q-Learning increased from 13.1% to 16.7%; NE-MDP increased from 11.3% to approximately 14.3%; the Shapley-NE-MDP model’s sensitivity started at 5.9% and slowly increased to approximately 7.3%, remaining the model with the lowest sensitivity, but its adaptability to changes in the difficulty of differential equation problems is significantly better than other models. Comparative experiments demonstrate that the Shapley-NE-MDP model has strong applicability to mathematical problems of varying difficulty.

To verify the generalization ability of the mathematical dynamic ensemble model based on Shapley-NE-MDP, mathematical dynamic ensemble models based on NE-MDP, Stackelberg-MDP, Q-Learning, and Shapley-NE-MDP were selected. 150 sets of samples were randomly selected from the InfiMM-WebMath-40B dataset and the MV-MATH dataset for repeated experiments. The fairness bias of the Shapley value of the model was calculated, as shown in Fig 13.

**Fig 13:**
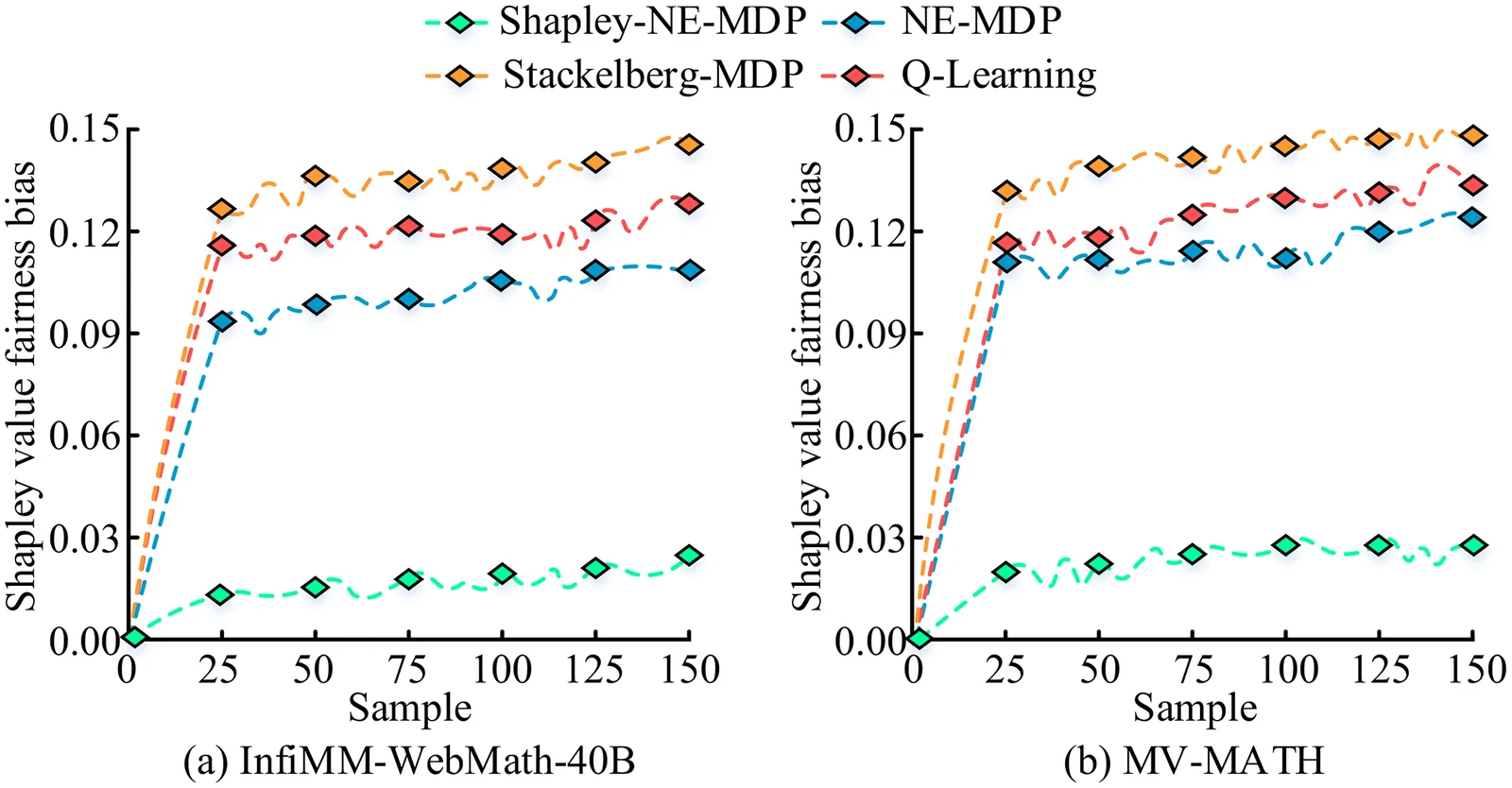
Comparison of Shapley value fairness bias across models.

In Fig 13(a), as the sample size increased from 25 to 150, the fairness bias of the Shapley value in the Shapley-NE-MDP model was approximately 0.012 with 25 samples, then slowly fluctuated and increased to approximately 0.024 with 150 samples. The fairness bias of the Shapley value in the NE-MDP model increased from 0.093 to 0.108, with some fluctuations but a generally stable trend. Q-Learning increased from around 0.116 to 0.123, showing a relatively obvious upward trend. Stackelberg-MDP increased rapidly from 0.126 to around 0.145, with the largest bias and a large fluctuation in the curve. In Fig 13(b), as the sample size increased from 25 to 150, the fairness bias of the Shapley value of the Shapley-NE-MDP model was approximately 0.019 with 25 samples, then steadily fluctuated and increased, reaching 0.027 with 150 samples, which is highly consistent with its performance on the InfiMM-WebMath-40B dataset. NE-MDP started at around 0.111 and fluctuated upwards within the range of [0.111, 0.124], with curve fluctuations similar to those on the InfiMM-WebMath-40B dataset. Q-Learning increased from around 0.117 to 0.133, showing a more pronounced upward trend. Stackelberg-MDP rose rapidly from 0.132 to around 0.149, exhibiting the largest bias and the largest curve fluctuation. Implementation results show that the Shapley-NE-MDP model has the smallest bias and the smoothest fluctuation among all models, demonstrating excellent fairness performance and good cross-dataset stability.

## 4 Discussion

Performance and application effect experiments verified the superiority of the Shapley-NE-MDP model, and its performance was significantly better than that of traditional mathematical prediction models. D. Bennet et al. proposed to combine the technology acceptance model with the random forest algorithm, and achieved accurate prediction by covering multiple dimensions such as technology complexity and financial dynamics. In comparison, the Shapley-NE-MDP model and this method had the same goal in multi-dimensional information integration, but this method focused on static feature mapping, and achieves prediction by screening key influencing factors and relying on the classification ability of random forest. It is suitable for scenarios with multiple factors but relatively smooth state evolution. The Shapley-NE-MDP model focused on dynamic optimization, quantifies the marginal contribution of multi-source information through the Shapley value method, and combined the stable policy characteristics of NE with the temporal state transition capability of MDP. It could handle complex dynamic decision problems where the system state iterates over time, and has stronger dynamic adaptability [28]. Y. Guo et al. proposed to mine the input-output relationship of variable efficiency devices through deep neural networks and construct a pipeline network delay dynamic model by combining the bilateral feature line method, which improved the modeling accuracy and scheduling scheme optimization effect. The research methods of the Shapley-NE-MDP model and this method are fundamentally different. The method used equivalent transformation to handle nonlinear problems, transforming the nonlinear activation function in the deep neural network into a mixed integer linear model to adapt to the linear optimization framework of the energy system. The Shapley-NE-MDP model adapted to nonlinearity through dynamic equilibrium, used the state transition probability of MDP to handle the nonlinear evolution of the system state, and combined the stable policy characteristics of NE to maintain policy stability in complex dynamic environments. For example, in tests of mathematical problems of different difficulties, its sensitivity was only 3.0%−7.3%, which is significantly lower than the comparative model [29]. The recursive physics-data integration algorithm proposed by W. Wei et al. integrated basic physical laws and data snapshots under the moving window framework, derived recursive algorithms to improve computational efficiency, and accurately captured the nonlinear and time-varying characteristics of horizontal dynamics. The Shapley-NE-MDP model and this method are similar in terms of dynamic modeling efficiency and multi-source information fusion, but the technical differences are significant. This method improved efficiency by optimizing the algorithm structure, abandoned the complete data snapshot, and used new data points within the moving window to update the transition matrix. The Shapley-NE-MDP model improved efficiency by optimizing the strategy and simplifying the path, reduced invalid strategy iteration by taking advantage of the stable strategy characteristics of NE, and reduced the cost of redundant information processing by combining the information filtering capability of the Shapley value method. In the test of 110 sets of experimental data, its training time was 26.5% less than that of the Shapley-NE model, and the CPU utilization rate was controlled at a low level of 78.5%. It achieved a balance between decision efficiency and resource consumption, and reserved sufficient time for emergency response of complex systems. It has important practical value in scenarios such as industrial fault early warning and dynamic resource scheduling [30].

In summary, the mathematical dynamic integration model based on Shapley-NE-MDP demonstrates significant performance, adaptability to dynamic changes across multiple scenarios, and strong application value. However, the research still has shortcomings in multi-scenario transfer adaptability. Future research could further expand the model’s application scenarios (such as industrial collaboration and public management) and optimize computational efficiency under large-scale multi-agent collaboration, providing more comprehensive technical support for dynamic decision-making problems in a wider range of fields.

## 5 Conclusion

To address the problems of imbalanced multi-source information value assessment, insufficient adaptability to dynamic environments, and poor decision stability in dynamic decision-making of complex systems, this study proposes a mathematical dynamic integration model that combines the Shapley value method, NE, and MDP. This model constructs a complete framework for multi-source information assessment, dynamic strategy optimization, and decision synergy, providing solid mathematical theoretical support and efficient algorithmic tools for complex system decision-making. Performance test results show that in dynamic optimization tests on the InfiMM-WebMath-40B and MV-MATH datasets, when the sample size is expanded to 150 groups, the increase in the number of model iterations is minimal (from 41 to 50, and from 44 to 53), with standard deviations increasing by only 0.010 and 0.011, respectively. In multi-model comparison experiments, the Shapley-NE-MDP model achieved an overall accuracy of 92.3%, which is 13.7%, 7.2%, 10.9%, and 16.1% higher than the Shapley-NE, NE-MDP, Stackelberg-MDP, and Q-Learning models, respectively. Its MAPE was as low as 3.8%, and its RMSE was only 2.1. Furthermore, in Gaussian noise interference experiments, the accuracy decrease was minimal under low, medium, and high noise levels (only increasing from 6.0% to 8.8% under high noise). These results validate the strong practical value of the Shapley-NE-MDP model in complex dynamic decision-making scenarios. It demonstrates outstanding performance in multi-source information evaluation accuracy, dynamic policy optimization capability, and decision robustness, providing a new method for dynamic integrated decision-making in complex systems, and possessing broader application prospects and significant practical implications.
